# Human Alveolar Echinococcosis—A Neglected Zoonotic Disease Requiring Urgent Attention

**DOI:** 10.3390/ijms26062784

**Published:** 2025-03-19

**Authors:** Ali Rostami, Britta Lundström-Stadelmann, Caroline F. Frey, Guido Beldi, Anja Lachenmayer, Bill C. H. Chang, Mohammad Mobin Norouzian, Andrew Hemphill, Robin B. Gasser

**Affiliations:** 1 Infectious Diseases and Tropical Medicine Research Center, Health Research Institute, Babol University of Medical Sciences, Babol, Iran; alirostami1984@gmail.com (A.R.); mobinnorouzian@gmail.com (M.M.N.); 2 Multidisciplinary Center for Infectious Diseases, University of Bern, Hallerstrasse 6, 3012 Bern, Switzerland; britta.lundstroem@unibe.ch (B.L.-S.); guido.beldi@insel.ch (G.B.); 3 Reference Laboratory of Federal Office for Food Safety and Veterinary Affairs, Institute of Parasitology and Echinococcus, University of Bern, Länggassstrasse 122, 3012 Bern, Switzerland; caroline.frey@unibe.ch; 4 Department for Visceral Surgery and Medicine, Bern University Hospital, University of Bern, 3010 Bern, Switzerland; anja.lachenmayer@insel.ch; 5 Department for Biomedical Research, Bern University Hospital, University of Bern, 3008 Bern, Switzerland; 6 Department of Veterinary Biosciences, Faculty of Science, The University of Melbourne, Parkville, VIC 3010, Australia; bill.chang@tigs.com.tw

**Keywords:** *Echinococcus multilocularis*, alveolar echinococcosis, biology, pathogenesis, diagnosis, treatment, prevalence, incidence, humans

## Abstract

Alveolar echinococcosis (AE) in humans is caused by the larval (metacestode) stage of *Echinococcus multilocularis*, commonly known as the ‘fox tapeworm’. This disease predominantly targets the liver and has an invasive growth pattern, allowing it to spread to adjacent and distant tissues. Due to its gradual progression and tumour-like characteristics, early diagnosis and prompt intervention are crucial, particularly as there are currently no highly effective vaccines or chemotherapeutics against AE. Current estimates suggest that ~10,500 new infections occur annually worldwide; however, more research is required to refine the prevalence and incidence data for both human and animal hosts in endemic areas of the world. This article discusses the biology of *E. multilocularis*, outlines aspects of the pathogenesis, diagnosis, treatment, and management of AE, reviews its global distribution, annual incidence, and prevalence, highlights the role of molecular parasitology in advancing therapeutic strategies, and presents recommendations for improving the prevention and control of AE in human populations.

## 1. Introduction

Tapeworms (cestodes) are significant multicellular parasites that infect vertebrate hosts worldwide, with species in the family Taeniidae—notably those within the genera *Taenia* and *Echinococcus*—being of particular socioeconomic and public health concern. Among these, *Echinococcus granulosus sensu lato* (s.l.) is responsible for cystic echinococcosis, whereas *Echinococcus multilocularis* causes alveolar echinococcosis (AE). Both diseases represent serious threats to human and animal health and have been classified as neglected tropical diseases (NTDs) by the World Health Organization (WHO) [1,2,3]. *E. multilocularis*, known as the ‘fox tapeworm’, is particularly noteworthy due to its ability to be transmitted from definitive hosts—primarily foxes, though other canids can also act as hosts—to humans, leading to an initially asymptomatic infection that progresses to chronic disease in intermediate and accidental hosts.

*E. multilocularis* is geographically restricted to the Northern Hemisphere, with its presence documented in continental Europe, Asia, and North America. Notably, China is estimated to contribute to >90% of the global AE burden in humans [4], potentially translating into tens of thousands of cases each year. While prevalence and disease burden estimates exist for some endemic regions [5], these figures might be unreliable due to the focal distribution of AE within transmission areas and a large proportion of asymptomatic cases that potentially remain undiagnosed, particularly in resource-limited and underserved populations. Furthermore, the absence of mandatory reporting for AE in many nations [6] contributes to a significant underestimation of disease incidence. Although AE does not pose a major public health risk in non-endemic countries—given that humans serve as dead-end hosts—accurate diagnosis and appropriate clinical management of this disease in immigrants from endemic regions remain essential. Surveillance and reporting efforts in endemic areas are also critical to enhancing assessments of disease burden and control strategies.

As of 2010, AE was responsible for 7771 deaths globally, with an estimated annual disease burden of ≥688,000 disability-adjusted life years (DALYs) [7]. In some regions, AE is increasingly recognised as an emerging disease [8,9,10,11]. Although comprehensive recent reviews of this disease remain scarce, available data suggest that its prevalence might be rising in parts of the Northern Hemisphere [2,12]. This trend may stem from improved surveillance in some countries, leading to greater case detection or from a reduced emphasis on sustained control measures, such as praziquantel-based baiting programs, aimed at reducing the prevalence of *E. multilocularis* infection in fox populations [13]. Despite being classified as a rare disease, AE can have devastating consequences, particularly in communities with limited healthcare access. In such settings, misdiagnoses (e.g., as advanced liver cancer) or delayed diagnosis can result in fatal outcomes [14,15,16].

This article aims to provide an overview of *E. multilocularis* and its biological characteristics, review salient aspects of the pathogenesis, diagnosis, and treatment of AE, outline its geographic distribution, incidence, and prevalence, examine how molecular insights into host–parasite interactions or parasitism can contribute to novel therapeutic developments, and summarise key components of the prevention and control of this neglected disease in human populations.

## 2. Life Cycle and Transmission of *E. multilocularis*

**Figure 1:**
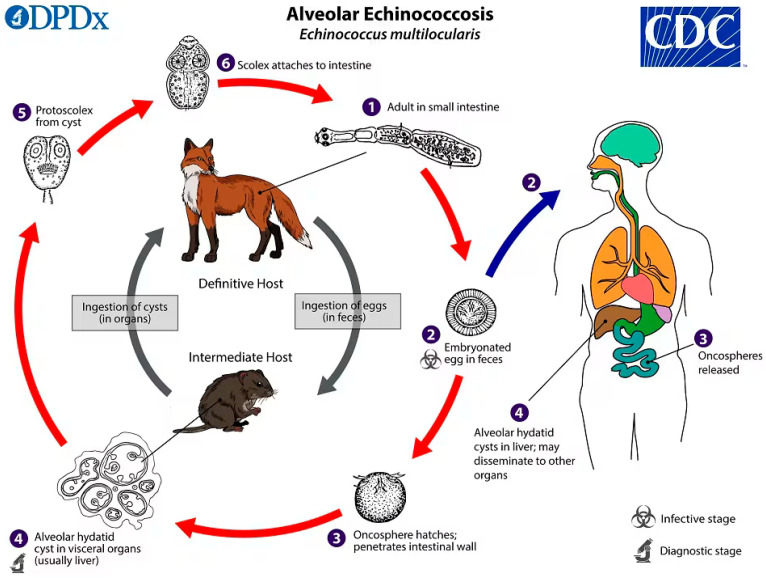
Life cycle of *Echinococcus multilocularis*. In its adult form, *Echinococcus multilocularis*—measuring about 1.2 to 4.5 mm—lives in the small intestine of its definitive host (1). Mature (gravid) proglottids release eggs that exit the host via faeces and are immediately infective (2). Once a suitable intermediate host ingests these eggs, they hatch in the small intestine, releasing six-hooked oncospheres (3). These oncospheres break through the intestinal wall and travel through the bloodstream to various organs—most often the liver. There, the oncospheres develop into thin-walled (alveolar) cysts that are multilocular (having multiple chambers) and expand outward by budding (4). Each of these cysts can generate numerous protoscoleces. A definitive (canid) host becomes infected when it consumes an intermediate host (or tissues thereof) that harbours these cysts and protoscoleces (5). After being swallowed, the protoscoleces emerge, attach to the lining of the small intestine (6) and mature into adults over a period of 32 to 80 days. Humans are considered accidental intermediate hosts. Infection occurs when a person ingests the eggs, which then release oncospheres in the small intestine (3); these oncospheres enter blood vessels and are passively transported via the blood stream primarily to the liver where they form cysts, causing alveolar echinococcosis (AE) (4). If protoscoleces are freed from the cysts, they may spread (metastasize) to other organs, such as the lungs, brain, heart, and/or bones, in a process sometimes referred to as secondary AE. Figure image originates from https://www.cdc.gov/dpdx/echinococcosis/index.html (accessed on 15 January 2025).

*E. multilocularis* follows a predator–prey transmission cycle, with canid species serving as definitive hosts and small mammals acting as intermediate hosts (Figure 1). The adult tapeworm, measuring between 1.0 and 4.5 mm in length, consists of 3–5 proglottids (segments) and resides in the small intestine of its definitive host. Among wild hosts, various fox species, golden jackals, coyotes, wolves, and raccoon dogs play key roles in maintaining transmission. Additionally, domestic dogs are highly susceptible and can serve as significant definitive hosts [17]. Gravid proglottids release eggs into the environment via host faeces. These eggs are subsequently ingested by appropriate intermediate hosts, which include numerous rodent species—particularly voles—as well as lagomorphs such as pikas and some insectivores [18]. The dominant intermediate host species involved in the sylvatic cycle varies depending on the geographical location.

Once ingested, the eggs travel through the digestive tract and hatch in the small intestine, releasing hexacanth oncospheres. These larvae penetrate the intestinal lining and are passively transported via the circulatory or lymphatic system to various organs. The liver is the primary site of infection, where the oncosphere transforms into a thin-walled larval stage (=metacestode or cyst) that proliferates asexually via external budding. Inside this larval stage, brood capsules develop, containing protoscoleces. When a definitive host consumes infected tissues from an intermediate host, the protoscoleces activate upon reaching the stomach, then evaginate and attach to the epithelial lining of the small intestine—embedding their scoleces within the intestinal crypts. Within ~25 days, they mature into adult egg-producing worms [19,20]. Humans as well as non-human primates and domestic dogs can act as accidental or ‘dead-end’ hosts [21], meaning that they do not contribute to parasite transmission but can still develop severe infection.

## 3. Pathogenesis and Clinical Progression of Disease

AE in humans arises from the accidental ingestion of *E. multilocularis* eggs, leading to the development of larvae in the liver. The outer surface of the larval stage is characterised by an acellular carbohydrate-rich laminated layer, which serves as a protective barrier. Beneath this layer, the parasite establishes a syncytial tegument with microtriches that extend into the laminated layer. This is followed by an inner germinal layer, which comprises connective and muscle tissues, glycogen reserves, neuronal cells, and crucially, stem cells. The larval stage contains fluid derived from the parasite and exhibits a multi-chambered infiltrative growth pattern. Although AE progresses gradually, it is a life-threatening condition if left untreated. A defining characteristic of *E. multilocularis* infection is the invasive nature of the larval stage, which leads to lesion formation and significant alterations in liver architecture [22,23].

**Figure 2:**
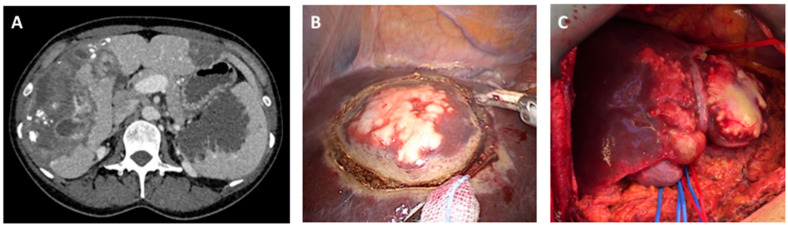
Pictorial summary of clinical and surgical aspects of alveolar echinococcosis (AE) in humans. (**A**) Computed tomography (CT) scan showing AE in the liver, spleen, and extraperitoneal areas. (**B**) An atypical laparoscopic resection of a lesion caused by AE from the liver dome. (**C**) AE in the left liver prior to left hemi-hepatectomy.

In the early stages, AE is typically asymptomatic, and clinical manifestations—such as hepatomegaly, abdominal discomfort, weight loss, fatigue and/or jaundice—may only emerge years after the initial infection, often coinciding with complications such as cholestasis or cholangitis. Radiologically and clinically, AE lesions can closely resemble malignant tumours (Figure 2), making accurate diagnosis difficult without the integrated use of multiple diagnostic methods. Given AE’s indolent progression and its striking similarities to malignancy, early detection and precise diagnosis are paramount. In some cases, AE exhibits a metastasis-like spread, with lesions extending from the liver to nearby structures in the abdominal cavity and via the bloodstream to distant organs, including the lungs, brain, bones, heart, and/or muscles (Figure 2). Chronic inflammatory responses contribute to progressive tissue damage and fibrosis [23]. Additionally, *E. multilocularis* excretes/secretes immunomodulatory molecules that enable the parasite to subvert host immune defences [24]. Clearly, a deeper understanding of AE pathogenesis is essential for improving clinical management strategies. Moreover, exploring the intricate host–parasite interactions at the immuno-molecular level may uncover novel therapeutic targets and new approaches for combating this challenging disease.

## 4. Recent Epidemiological Insights into the Incidence and Prevalence Trends of Human AE

**Figure 3:**
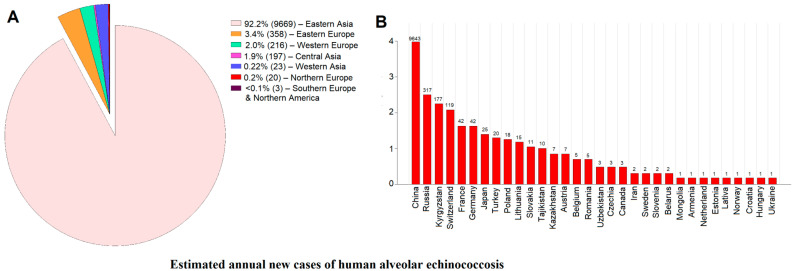
Estimated number of new cases of human alveolar echinococcosis (AE) annually by region and country. (**A**) Global distribution of new cases of AE in humans in known endemic regions in the Northern Hemisphere. Globally, the annual number of AE cases is estimated at 10,489 per year (range: 8191–14,409), with the majority of cases in Eastern Asia (predominantly in China), Eastern Europe (mainly in Russia), Central Asia (mainly in Kyrgyzstan), and Western Europe (mainly Switzerland, Germany, and France), as well as ~50 new cases reported annually in other endemic regions; (**B**) estimated numbers of new cases of human AE annually by country for endemic regions. A logarithmic scale is used for the y-axis to account for differences between high- and low-incidence areas. The highest estimated case numbers are in China and Russia, while some European and North American countries report lower numbers. Data are summarised from ref. [25].

A recent detailed analysis of publicly available datasets [25] estimated the global, regional, and national annual incidence rate (AIR) and prevalences of human AE in 47 countries—all in the Northern Hemisphere. The estimated global median number of AE cases was 10,489 per year (range: 8191–14,409), significantly lower than previous estimates [26]. Notably, 92.2% of cases occurred in Eastern Asia (predominantly in China), with ~850 cases reported annually in other endemic countries (Figure 3A).

The estimated numbers of new cases of human alveolar echinococcosis (AE) annually by region/country are given in Figure 3B. China appears to be the most affected country, with an estimated pooled prevalence of 0.86% (95% CI: 0.78–0.94%), reaching 1.94% in Sichuan and 1.90% in Qinghai, whereas Tibet, Gansu, Ningxia, and Xinjiang reported lower prevalences [25]. The estimated annual incidence rate (AIR) was 0.7 per 100,000, corresponding to 9643 new cases annually (range: 7472–13,408). These findings indicate a 42% decline in AE cases compared with previous estimates (9643 vs. 16,629 cases) [25], although the disease burden remains substantial in endemic provinces such as Qinghai and Sichuan.

In Central Asia, the incidence of AE has risen significantly over the past two decades [25]. In Kyrgyzstan, for instance, cases increased from 0–3 annually (1996–2003) to 140–200 per year (2013–2016), with an estimated AIR of 2.62 per 100,000 and 177 new cases annually (range: 164–188). In Kazakhstan, 135 cases were reported between 1996 and 2019, corresponding to an AIR of 0.037 per 100,000. In Turkey, 641–918 cases were reported between 1939 and 2018, with ~20 new cases per year and an estimated AIR of 0.023 per 100,000 [25].

In Canada, the number of confirmed AE cases has more than tripled over the past two decades, with 12 cases (2000–2014, AIR: 0.002 per 100,000) and 17 cases (2014–2020, AIR: 0.007 per 100,000) recorded [25]. Additionally, a single case was reported in Ontario in 2024 [27]. In the USA, AE remains exceedingly rare, although two new cases were reported in Vermont in 2020 and 2022, both presenting with liver and lung lesions, with molecular analysis confirming a European haplotype of *E. multilocularis* [28,29].

In Europe, *E. multilocularis* is widespread in wildlife, but the number of human cases varies considerably by region. France, which maintains a national AE registry, recorded 944 confirmed cases between 1972 and 2021, with an AIR of 0.062 per 100,000 (2013–2021) and 30–57 new cases annually [25]. In Switzerland, the AIR increased from 0.15 per 100,000 (1956–2000) to 0.26 per 100,000 (2001–2005), with a sharp rise in reported cases in 2020 (98 cases) and 2021 (140 cases) [25]. In Germany, the prevalence was estimated at 0.71%, with an AIR of 0.05 per 100,000, showing an increasing trend in recent years [25]. AE is also gradually increasing in Eastern and Northern Europe, with Lithuania having the highest reported AIR (0.52 per 100,000), followed by Estonia, Latvia, Norway, and Sweden (0.056, 0.063, 0.02, and 0.024 per 100,000, respectively). In Russia, a review of 117 data sources (1996–2023) identified 7933 confirmed cases, with an estimated AIR of 0.22 per 100,000 and 317 new cases annually [25]. These figures are lower than previous estimates [26], which relied on model-based projections rather than confirmed case reports, possibly leading to an underestimation in the recent analysis [25]. AE incidence also appears to have increased in Eastern Europe, including Ukraine (AIR: 0.002 per 100,000), Czechia (0.026 per 100,000), Hungary (0.011 per 100,000), Poland (0.047 per 100,000), and Slovakia (0.2 per 100,000) [25].

## 5. Comprehensive Diagnosis of Disease Requires a Multimodal Approach

AE must be considered in patients from endemic areas, particularly when characteristic radiological abnormalities are detected. Individuals with a history of exposure to definitive hosts, such as canids, may be at an increased risk of infection [27]. However, diagnosis is often delayed or occurs incidentally, given the prolonged asymptomatic phase, which can span 5 to 15 years. Due to the chronic nature of the disease and the prolonged treatment required, AE also imposes a significant psychological burden on affected individuals [28,29]. Following an initial clinical assessment, multiple diagnostic techniques are commonly employed [30,31,32]. Imaging remains central to AE detection, with three primary modalities used: (i) ultrasonography (US), which typically reveals multi-vesicular hepatic lesions; (ii) computed tomography (CT), which provides detailed insights into the lesion distribution, involvement of adjacent structures, and possible metastasis; and (iii) magnetic resonance imaging (MRI), which enhances the representation of the bile duct and vascular involvement. These imaging findings are used to classify lesions according to the PNM system, where P represents the parasitic mass in the liver, N denotes the extent of extrahepatic spread, and M indicates the presence or absence of distant metastases [31]. Serological tests, such as the enzyme-linked immunosorbent assay (ELISA) and immunoblotting, are used to detect serum antibodies against *E. multilocularis* antigens (e.g., EmII/3-10 and Em18). However, the sensitivity and specificity of these assays can vary, depending on factors such as the disease stage, co-infections, or underlying health conditions [32,33,34]. Molecular methods, particularly polymerase chain reaction (PCR)-based assays, enable the precise identification of *E. multilocularis* DNA in tissue samples, particularly following the surgical removal of lesions. DNA sequencing can further confirm species identity [32]. Despite their utility, invasive procedures, such as fine-needle aspiration and biopsy, pose risks, including potential complications and parasite dissemination. Tissue samples can also undergo histopathological examination using techniques such as periodic acid–Schiff staining or immunohistochemistry to detect larvae [23,30,35,36]. Immunohistological staining with monoclonal antibodies, such as Em2G11, enables the detection of small particles of *E. multilocularis* (designated as SPEMs), which represent microscopic parasite outgrowths within surrounding tissues, including lymph nodes [37]. Given the complexity of AE, an accurate diagnosis often requires the combined use of imaging, serology, and molecular and histopathological approaches. The interpretation of findings should be conducted by experienced clinicians and pathologists, as a multidisciplinary approach is essential to ensuring a timely and accurate diagnosis.

## 6. Clinical Management of Human Disease—Surgical and Medical Interventions

The most effective curative approach for AE is radical surgical excision of the parasitic lesion—an option pursued in ~20–50% of cases [38]. Given the invasive nature of *E. multilocularis*, complete removal of the parasitic mass is recommended whenever feasible. Although liver transplantation was historically considered for advanced AE, the risk of rapid disease progression due to immunosuppression makes this approach undesirable, despite its continued mention in WHO guidelines [39].

For cases where surgery is not possible, or as an adjunct to surgical intervention, chemotherapy with benzimidazoles remains the standard of care. Albendazole and mebendazole are commonly used agents, administered orally over prolonged periods, often for several years or even lifelong [33,40]. The primary mode of action of these drugs is believed to involve inhibition of microtubule polymerisation by binding to beta-tubulin, although direct experimental confirmation in *E. multilocularis* is lacking. Additionally, these drugs impair glucose uptake, leading to glycogen depletion and structural damage in the parasite’s germinal layer, ultimately resulting in cellular autolysis [41,42]. Despite their efficacy in suppressing the proliferation of the larval stage in the affected host, benzimidazoles do not eliminate the parasite entirely, as they exhibit parasitostatic rather than parasiticidal effects [33,40]. This limited efficacy has been linked to stem cells of the larval stage, which express a beta-tubulin isoform (Tub-2) that does not effectively bind these drugs, along with restricted drug uptake and a short half-life [43].

Of the two benzimidazoles used, albendazole is preferred over mebendazole due to a higher bioavailability, although mebendazole remains an alternative if albendazole is unavailable or poorly tolerated. Treatment duration is determined based on lesion size, location, disease progression, and the patient’s overall response [30]. Standard dosing regimens for AE involve significantly higher doses than those used for other parasitic infections, contributing to substantial treatment costs. Albendazole is typically administered at 10–15 mg/kg body weight twice daily (max. 20 mg/kg), while mebendazole requires 40–50 mg/kg three times daily [30,31]. Since fat-rich meals enhance benzimidazole absorption, dietary adjustments may be recommended, and drug levels can be monitored in blood samples to ensure appropriate dosing while minimising toxicity risks [31,44]. However, given the lack of alternative treatments, therapeutic monitoring is usually reserved for select cases. Adverse effects are relatively common, with a German cohort-study reporting general side effects in 49.3% of patients and severe toxicity in almost 7% [39]. These challenges highlight the urgent need for new, more effective, and safer treatment options for AE.

Throughout chemotherapy, regular imaging assessments (US, CT, MRI) are critical to monitor lesion progression and evaluate treatment response. Following chemotherapy, serological testing (e.g., anti-Em18 antibodies) can help assess sero-reversion, while imaging modalities, such as ^18^F-FDG PET-CT, MRI or contrast-enhanced ultrasound, might be used to detect active disease [31,45,46,47]. In cases where hepatectomy is performed, post-surgical adjuvant benzimidazole therapy and follow-up monitoring via imaging and serology are often recommended, particularly when preoperative anti-Em18 antibody levels suggest ongoing infection [48]. However, major surgical procedures carry inherent risks, including postoperative haemorrhage, hepatic failure, portal hypertension, biliary complications, abscess formation, and/or secondary echinococcosis.

Given the complex nature of AE, patient management must be highly individualised and requires a multidisciplinary approach. Decisions regarding surgical intervention and medical treatment should be made by specialists with expertise in echinococcosis, carefully considering the patient’s immune status, disease stage, and overall prognosis [49].

## 7. Insights into Host–Parasite Interactions at the Molecular Level—Implications for Novel Interventions

The first draft of the *E. multilocularis* nuclear genome (115 Mb) was published in 2013 [50]. This study provided foundational insights into the evolution of parasitism, revealing synteny with relatively closely related cestodes and distantly related blood flukes (schistosomes). Compared with many animal species, *E. multilocularis* exhibits a significant loss of genes associated with fundamental molecular and biochemical pathways, including homeobox genes and key determinants of stem cell fate. As a result, this parasite relies on host-derived nutrients (e.g., fatty acids, cholesterol, purines, pyrimidines and amino acids), unique detoxification mechanisms, and an expansion of non-canonical heat shock proteins and other molecules that mediate host–parasite interactions [50]. More recently, transcriptomic analyses of *E. multilocularis* larval vesicles and germinal layer cells derived from in vitro-cultured parasites identified ~1180 genes linked to stem cell function, providing a valuable foundation for developmental biology and drug discovery research [51].

The availability of the *E. multilocularis* genome [50] has facilitated fundamental research into the molecular and biochemical pathways, developmental processes, and host–parasite interactions of this species. However, sequencing efforts should be expanded to include genomes from additional global isolates using advanced methods [52,53,54,55]. The inclusion of multiple genomes and transcriptomes covering key developmental stages and tissues would significantly enhance gene annotation, improve the accuracy of genetic mapping, and facilitate molecular and biochemical explorations. Moreover, comparative genomic analyses could aid in identifying novel drug targets, particularly parasite-specific genes with no orthologs in host species. Further, large-scale genomic investigations would also enable the assessment of genetic variation within and between *E. multilocularis* populations, which is critical for understanding host–parasite interactions, immune evasion strategies, and transmission patterns [56]. These insights could ultimately support the development of targeted intervention strategies.

The establishment of in vitro culture systems for *E. multilocularis* [51,57,58,59,60] presents new opportunities for functional genomic studies, allowing researchers to investigate gene essentiality [61] through gene knockdown [62,63] and potentially adapt knock-out and knock-in strategies already developed for trematodes [64,65,66,67]. However, caution is necessary when interpreting results, as in vitro conditions differ significantly from those in vivo. Recent advances in lentivirus- and CRISPR/Cas9-based functional genomic technologies, successfully applied to trematodes [68,69,70,71,72], could be adapted for use in *Echinococcus* species. Integrating in vitro culture of larvae with genomics, transcriptomics, proteomics and metabolomics could accelerate the development of new therapeutics by identifying key parasite-specific pathways and molecular targets for intervention.

A drug screening platform has been established to evaluate compounds from curated chemical libraries against *E. multilocularis* metacestodes [73,74,75,76]. Various assessment criteria include physical damage to larvae [74], vesicle viability [76,77], germinal layer cell survival [76], larval formation rate in vitro [78,79], and protoscolex motility [80]. These techniques support structure–activity relationship (SAR) analyses [81,82,83], allowing for the systematic refinement of promising drug candidates. Complementary techniques, such as electron microscopy, affinity chromatography for drug-binding protein identification, and mitochondrial respiration assays, further enhance drug discovery efforts [81,82,83]. Compounds demonstrating selectivity and a favourable therapeutic window (low toxicity in mammalian cells) are then tested in standardised AE mouse models to assess efficacy [73,81,84,85].

Drug repurposing has emerged as a promising avenue for identifying new effective AE treatments [86,87,88]. Given the tumour-like characteristics of *E. multilocularis* metacestodes, anti-cancer drugs were initially explored for therapeutic potential. While some compounds demonstrated in vitro activity, none exhibited sufficient in vivo efficacy, and many were associated with severe side effects [39]. Screening of a broader panel of anti-infective agents in both in vitro and AE–mouse models identified amphotericin B [89,90,91], nitazoxanide [74,92], and mefloquine [93] as promising candidates. Additionally, some natural compounds have been evaluated [84,94,95,96,97,98], though none has yet demonstrated strong efficacy. Continued research in genomics, drug discovery, and host–parasite interactions will likely be essential for advancing novel therapeutic strategies to combat AE effectively.

## 8. Concluding Remarks—Need to Strengthen Surveillance and Control of a Neglected Disease

The burden of AE is considerable, and reports suggest that its incidence has risen over the past decade, likely due to increased environmental exposure to *E. multilocularis* eggs from infected definitive hosts [8]. In Switzerland, for example, human AE cases increased following fox population growth after rabies eradication [99]. Since foxes and other canids, such as coyotes, frequently inhabit urban areas, contamination of backyards and gardens with parasite eggs is a persistent risk [100,101]. Domestic dogs contribute significantly to transmission, with estimates indicating that they account for 4–19% of environmental contamination in Central Europe [102]. The rising incidence of AE in both humans and dogs in Switzerland suggests that contamination with *E. multilocularis* eggs is increasing [5,21,103,104]. However, significant gaps remain in our understanding of transmission dynamics, ecological factors, and genetic variation within *E. multilocularis* populations.

Surveillance programs help monitor AE prevalence, although implementation varies. Mass ultrasound screening has been effective for assessing infections in endemic populations [10]. China has established active surveillance programs [105], and Kyrgyzstan relies on passive reporting [106]. In developed regions, low AE prevalence may reflect heightened awareness and early diagnosis, but this is unlikely in remote resource-limited communities, where cases often go undetected. In these settings, infection rates in domestic dogs can reach 10–20% [107,108], much higher than the ~1% prevalence in canines in Central Europe [109]. Since humans interact with domestic dogs far more frequently than with wild foxes, transmission is likely amplified in high-risk communities.

Control efforts primarily focus on interrupting transmission by reducing infection sources in foxes, dogs, and other definitive hosts. Monthly praziquantel treatment of domestic dogs remains the most effective way to prevent environmental contamination [13]. In controlled settings, praziquantel-laced baits have successfully decreased the prevalence of *E. multilocularis* infection in foxes [110], though this method is impractical across large or remote areas [13,102]. Public education campaigns are vital in raising awareness about infection risks, hygiene practices, and food safety [13]. Although controlling AE in domestic dogs is feasible, wild canids remain a major transmission reservoir, complicating efforts to fully break the lifecycle of *E. multilocularis*.

Preventing AE in humans requires minimising the ingestion of *E. multilocularis* eggs through contaminated food, water, or soil. A major challenge is detecting viable eggs, as PCR assays confirm parasite presence but cannot determine viability or infectivity [111]. In developed areas, AE is managed through surgical intervention or long-term chemotherapy, but both approaches are costly and psychologically burdensome. In under-resourced regions, limited healthcare-access results in many undiagnosed and untreated cases. Expanding medical infrastructure and treatment availability is critical. Moreover, there is also a need for new more effective drugs, which might be developed through proteomics and metabolomics, for example, to better understand parasite biology and host interactions [112,113,114].

Education remains a cornerstone of prevention, particularly in high-risk areas where community awareness of AE is limited. Sustained public health campaigns and surveillance programs will be essential to reducing transmission, improving early diagnosis, and ensuring better treatment access. With continued research and public health efforts, it might be possible to substantially lower AE prevalence and its impact in endemic regions.
